# Factors influencing the implementation of severe acute malnutrition guidelines within the healthcare referral systems of rural subdistricts in North West Province, South Africa

**DOI:** 10.1371/journal.pgph.0002277

**Published:** 2023-08-18

**Authors:** Faith Nankasa Mambulu-Chikankheni

**Affiliations:** 1 Department of Curriculum and Teaching Studies (Human Ecology), Nalikule College of Education, Lilongwe, Malawi; 2 Centre for Health Policy, School of Public Health, University of the Witwatersrand, Johannesburg, South Africa; African Population and Health Research Center, KENYA

## Abstract

Severe acute malnutrition (SAM) is associated with 30.9% of South Africa’s audited under-five children deaths regardless of available guidelines to reduce SAM at each level of a three tyre referral system. Existing research has explored and offered solutions for SAM guidelines implementation at each referral system level, but their connectedness in continuation of care is under-explored. Therefore, I examined implementation of SAM guidelines and factors influencing implementation within subdistrict referral systems. An explanatory qualitative case study design was used. The study was conducted in two subdistricts involving two district hospitals; three community health centres, four clinics, and two emergency service stations. Between February to July 2016 and 2018, data were collected using 39 in-depth interviews with clinical, emergency service and administrative personnel; 40 reviews of records of children younger than five years; appraisals of nine facilities involved in referrals and observations. Thematic content analysis was used to analyse all data except records which were aggregated to elicit whether required SAM guidelines’ steps were administered per case reviewed. Record reviews revealed SAM diagnosis discrepancies demonstrated by incomplete anthropometric assessments; non-compliance to SAM management guidelines was noted through skipping some critical steps including therapeutic feeding at clinic level. Record reviews further revealed variations of referral mechanisms across subdistricts, contradictory documentation within records, and restricted continuation of care. Interviews, observations and facility appraisals revealed that factors influencing these practices included inadequate clinical skills; inconsistent supervision and monitoring; unavailability of subdistrict specific referral policies and operational structures; and suboptimal national policies on therapeutic food. SAM diagnosis, management, and referrals within subdistrict health systems need to be strengthened to curb preventable child deaths. Implementation of SAM guidelines needs to be accompanied by job aids and supervision with standardised tools; subdistrict-specific referral policies and suboptimal national policies to ensure availability and accessibility of therapeutic foods.

## Introduction

Developing guidelines for managing severe acute malnutrition (SAM) among under-five children has been a global focus since the 1980s; however, the condition remains a public health concern [1–4]. SAM accounted for approximately 17 million incidences among this age group, while malnutrition-related factors accounted for 45% of 5.3 million deaths in 2018 globally [5]. Approximately 4.2 million SAM incidences in 2018 were from Sub-Saharan African countries, partly due to a combined HIV and TB burden [6–8]. South Africa (SA) has adopted SAM management guidelines; however, 30.9% of all audited under-five children were associated with SAM in 2017 [9].

Studies on the implementation of SAM guidelines report experiences at community, clinic, and hospital levels separately despite their duty to offer a continuum of care through operating within a referral system [10–12]. Integrated Management of Childhood Illness (IMCI) informs the assessment, classification and management of moderate acute malnutrition (MAM), and the immediate management (stabilisation) of young children who present with SAM at primary healthcare (PHC) facilities [13]. SAM diagnosis involves consecutively assessing three indicators; however, preference for one indicator led to misdiagnosis which later led to prescribing treatment for a severe condition when a case is a moderate one or prescribing treatment for a moderate condition when a case is a severe one [11,14–16]. For instance, comprehensive SAM diagnosis in a Ghanaian hospital reduced admission which is meant for treating severe conditions from 81 to 39 due to excluding cases which were moderate [14]. In separate instances, erroneous classification of SAM led to the missing of 50% of SAM cases in Gambia and poor stabilisation of cases at PHC facilities in SA [11,17]. Such errors in IMCI implementation are attributed to incompetence from poor training, lack of supportive supervision and resources [11,17,18].

SA studies focus on referral systems functionality by proposing a seamless continuation of care and the need to link community-based services to facilities; however, they seldom explore SAM care during the referral process [19,20]. At hospitals, standard SAM treatment included in SA guidelines are the WHO 10 steps which have proven feasible and capable of reducing SAM fatality in rural SSA settings [6,14,21]. However, errors, inconsistency and not implementing some of the guidelines emanating from poor clinical skills, lack of resources or supervision impede the guidelines’ potential to reduce inpatient SAM deaths [14,20,22–25]. There is a need to explore whether SAM cases are managed based on standard guidelines at all levels of the referral system along with related factors.

To achieve SDG 3.2, efforts to reduce preventable deaths of under-five children, SSA should strive to identify and address bottlenecks to the successful implementation of SAM management guidelines [5,26]. In previous studies, the limitations of managing SAM at the SA community level have been identified [27] and the comprehensiveness of SA’s SAM guidelines has been appraised [28]. To enhance the narrative, this study explored the complexity of implementing SAM guidelines by focusing on the diagnosis, management, and referral of under-five children within the rural subdistrict’s referral system and the factors influencing these practices.

## Materials and methods

### Study design

This study employed an explanatory qualitative case study design [29]. Using a qualitative approach enabled the holistic exploration of the complexity of implementing SAM guidelines [30,31] within the study context. The chosen study design enabled the exploration of the ‘how’ and ‘why’ phenomena underpinning the implementation of SAM guidelines within the selected subdistrict referral levels [32,33]. The explanatory aspect of the design enabled a detailed description of concepts underlying implementation for practical understanding rather than providing conclusive evidence [29,34].

### Description of the study setting

North West Province is to the west of Gauteng Province and south of Botswana, the province is divided into four main district municipalities. It was selected for this study because it had the second-highest incidence of SAM in South Africa (registering 8.1 cases per 1000 under-five children) during this study’s conceptualisation in 2015 [35]. Two rural subdistricts were selected using the principle of maximum variation, which enables the inclusion of a wide range of individuals, groups or settings for multiple perspectives that exemplify the complexity of the real-world [36]. One subdistrict registered the highest incidence of 19.5 cases per 1000 compared to the national average of 4.5 [35]. The other subdistrict was implementing the national health insurance (NHI) interventions as a means of strengthening PHC services. NHI interventions include healthcare referral strengthening strategies, contracting general practitioners for clinics, community referral using mobile technology and establishing district clinical specialist teams (DCSTs) to enhance clinical governance [37]. The assumption was that the subdistrict implementing interventions had the potential to illustrate the role of NHI interventions in influencing SAM care. Henceforth, these subdistricts will be referred to as NHI and non-NHI subdistricts.

### Sampling

Nine facilities were selected based on their role in the referral process and proximity to a referral centre. Each subdistrict had one referral hospital, emergency services station and NHI subdistrict had one Community health centre (CHC); thus these five facilities were selected by default. Table 1 depicts the selected nine facilities per level of care in each subdistrict.

**Table 1:** Study facilities per subdistrict.

Level of care	NHI Subdistrict	Non-NHI Subdistrict
Subdistrict hospital	1	1
Emergency service station	1	1
Community health centre (CHC)	1	2
Clinic	2	2

Forty records for children aged between six months and five years, who were treated and referred for SAM between January and June 2016, were identified using admission registers at hospitals and referral registers at clinics. Based on availability, there were 19 records (patient files and child health records (Road to Health Books) at PHC facilities and 21 records (patient files) at the subdistrict hospitals were solicited. Records were difficult to trace at the hospital level because of a mismatch between registers and the filing system; thus only Road to Health Books (RHBs) with files of children admitted at Non-NHI hospital were available.

Purposive quota sampling, which allowed the inclusion of various categories of individuals involved in the diagnosis, management, and referral of SAM cases was used to recruit 39 participants for in-depth interviews. The variety of IDI participants is depicted in Table 2.

**Table 2:** In-depth interview participants.

Category	NHI	Non-NHI	Total
Managers	6	6	12
Doctors	2	2	4
Nurses	7	10	17
Dieticians	1	1	2
EMS personnel	2	2	4
**Total**	**18**	**21**	**39**

The doctors and emergency services personnel were recruited based on their willingness and availability to participate, nurses were recruited until saturation was reached [38], and two dieticians by default because each district hospital had one.

Convenience sampling was used to recruit nurses and doctors who were observed while implementing SAM guidelines during consultations or ward rounds during the researcher visits between February to July 2016.

### Data sources and collection

Data were collected from February to July of 2016 using (i) *facility appraisals*; (ii) *patients’ record reviews;* (iii) *in-depth interviews;* and (iv) *non-participatory observations*. Facility appraisals audited the availability of resources required for SAM care or referrals in facilities and emergency service stations. Aspects appraised included the availability and quality of infrastructure, human resources, supplies and transportation services. Forty patient records were reviewed using a structured data extraction form which captured whether required SAM diagnosis, management and referral indicators were implemented. The extraction was crosschecked by a doctor, nurse and dietician in the study setting to ensure the captured data was correct. In-depth interviews were conducted in English, using interview guides with questions on how participants implemented SAM diagnosis, management and referral guidelines as well as whether implementation was enabled or hindered by their context or other aspects. All interviews were audio-recorded and lasted between 40 to 60 minutes. For convenience and privacy, interviews were conducted during participants’ breaks in empty wards, offices or unused consultation rooms. Participants were recruited after two researchers (FNM-C and JE) introduced the study to potential participants. Observations were conducted using a checklist capturing practices involved in SAM diagnosis, management and referral. Transit practices were not observed due to the absence of EMS-referred SAM cases during researcher visits.

### Data analysis

In-depth interviews were transcribed verbatim and thematic content analysis was used to analyse the data from the transcripts, observational notes and facility appraisals. This analysis involved reading data sources (interview transcripts, observational notes, and facility appraisals) line-by-line, coding, extracting emerging sub-themes and then, main themes [39], using NVivo software. The themes derived were then aligned to SAM diagnosis, management and referral practices as well as factors that influenced practices. The patient record entries were analysed per frequency of implementing SAM guidelines steps. This involved extracting the implementation of each guideline’s steps from the files to elicit whether all steps were administered per case reviewed.

### Methodological rigour

Subdistrict presentations took place in February and May 2018 to allow participants to discuss, endorse, contradict, and add any other relevant information to conclusions made by the researchers. Furthermore the findings were assessed by two paediatricians in academia who made suggestions on how to best present clinical aspects. The rigour of all data was ensured by triangulation. For instance, interviews were triangulated with observations and record reviews as well as eliciting information from various cadres of the SAM management referral continuum.

### Ethical considerations

Ethical approval was obtained from the University of the Witwatersrand’s Human Research Ethics Committee (Medical) referenced M151015 and the North West Provincial Department of Health. Before data collection, written informed consent to conduct and record interviews was obtained from facilities and participants. Pseudonyms are used to conceal participants’ and facilities’ identities.

## Findings

Findings are presented on themes, namely i) discrepancies in SAM diagnosis, ii) limited adherence to feeding guidelines, iii) inconsistent adherence to SAM management steps, and iv) unavailable and inconsistent documentation. To contextualise the study settings, Table 3 depicts the attributes of facilities based on facility appraisals.

**Table 3:** Study facilities and attributes.

Level of care	NHI	Non-NHI
***Hospitals***	***NHI Subdistrict hospital*** • 4-bed paediatric ward • 5 general doctors (for all departments) • 4 general nurses • 1 paediatric ward manager (general nurse) • 1 dietician	***Non-NHI Subdistrict hospital*** • 22-bed paediatric ward • 2 general doctors (for paediatric ward) • 6 paediatric nurses • 1 paediatric ward manager (paediatric nurse) • 1 dietician
***Community Health Centres (CHC)***	**NHI CHC** • 4 Km to NHI hospital • Modern and big facility (sickbay for children) • 1 doctor • 4 general nurses (1 community service) • 2 managers (general nurses)	***Non-NHI CHC 1*** • 24 Km to non-NHI hospital • Old and small facility (no sickbay for children) • No doctor • 3 general nurses • 1 manager (also serving as a nurse)
		***Non-NHI CHC 2*** • 12Km to non-NHI hospital • Modern and big facility (sickbay for children) • 1 doctor • 4 general nurses • 2 managers (general nurses)
***Clinics***	***NHI Clinic 1*** • 0.5Kms to NHI hospital • Small facility and old (child wing in container structure) • 1 doctor • 2 general nurses for children wing (1 on community service) • 1 manager (general nurses)	***Non-NHI Clinic 1*** • 5Kms to non-NHI hospital • Small and modern facility (general sickbay) • 3 general nurses (2 community service) • 1 manager (also serving as a nurse)
	***NHI Clinic 2*** • 47Kms to NHI hospital • Small and modern facility • Visiting doctors (once a week) • 2 general nurses (for all departments) • 1 manager (also serving as a nurse)	***Non-NHI Clinic 2*** • 108Kms to Non-NHI hospital • Small and old facility (general sickbay) • No doctor • 3 general nurses (serving all departments) • 1 manager (also serving as a nurse)
***Emergency Services***	***NHI Emergency Service*** • 3Kms to NHI hospital • 2 EMS personnel • 2 high technology and 2 basic ambulances	***Non-NHI Emergency Service*** • 23Kms to NHI hospital • 2EMS personnel • 2 high technology and 2 basic ambulances

### Discrepancies in SAM diagnosis

SAM diagnosis was incomplete at PHC facilities and hospitals. Table 4 portrays observed discrepancies and associated factors.

**Table 4:** Themes on SAM diagnosis.

Broad Themes	Practices Sub-Themes	Factors Sub-Themes
Discrepancies in SAM diagnosis	◾ Incomplete anthropometry at PHC and hospital levels	◾ Poor clinical skills ◾ Unavailability of height and length measuring equipment ◾ Irregular and unstructured monitoring of SAM practices
	◾ Variations in SAM diagnosis amongst different cadres of health workers	◾ Same as above ◾ Limited opportunity for real-time consensus amongst different cadres

Patients’ records revealed incomplete SAM diagnosis in all subdistricts based on incomplete anthropometric assessments depicted in Table 5.

**Table 5:** Incomplete anthropometry for 19 PHC and 21 hospital cases.

Anthropometric Indicator	PHC Level (19 records)	Hospital Level (21 records)	Total (40 records)
• Weight • Height • MUAC • Oedema • Blood glucose	• 19/19 (100%) • 0/19 (0%) • 3/19 (16%) • 2/19 (11%) • 0/19 (0%)	• 21/21 (100%) • 3/21 (14%) • 11/21 (62%) • 8/21 (38%) • 1/21 (5%)	• 40/40 (100%) • 3/40 (8%) • 14/40 (35%) • 10/40 (20%) • 1/40 (3%)

The required weight for height, MUAC and oedema indicators were missed due to high concentration on weight for age and other complications:

"I check weight for age and complications like hypoglycaemia, be it sepsis, be it severe anaemia or dehydration" *(IDI 2B*, *Doctor*).

Only dieticians assessed all required indicators leading to differences with other cadres of health workers in classifying SAM.

**File 1****: *Doctor Diagnosis—***classified the illness as SAM based on assessing weight for age and blood glucose. ***Dietician Diagnosis—***opposed the doctor’s diagnosis by writing “Not SAM based on the findings from assessing weight for age, weight for length, MUAC, oedema and blood sugar".

Only 11/21 SAM assessments done at the hospital level were crosschecked by dieticians who opposed 4/11 SAM diagnoses made by doctors (in files 1A, 3A, 1B, 3B). Additionally, 13/14 MUAC assessments of the 40 records were conducted by dieticians.

### Factors influencing discrepancies in SAM diagnosis

#### Poor clinical skills and unavailability of assessment equipment

Diagnosis discrepancies partly resulted from a lack of skills emanating from inadequate SAM training by general doctors and nurses.

“Nurses don’t have the capacity to screen malnutrition because there are no dieticians to guide them… they adequately check growth charts like underweight for age, but the dietician is needed to train them on other things” *(IDI 7A*, *Doctor)*.

“…the doctors are not formally trained [in-service] on SAM; as a result, they don’t always diagnose and treat correctly…” *(IDI 3A*, *Dietician)*.

The lack of skills due to the absence of in-service training coincided with the unavailability of height boards and length mats at PHC facilities, thus, further limiting the capacity for comprehensive SAM assessments.

#### Irregular and unstructured monitoring

Lack of structured, regular and regulated monitoring also contributed to inconsistent SAM diagnosis. The NHI hospital manager reported monitoring practices, but other facility managers rarely or poorly monitored practices:

“We track their [PHC nurses] performance on an annual basis…” *(IDI 10A*, *PHC facility manager*).

"I do not assess IMCI use; however, in 2009, nurses from the district came to assess IMCI use…they were looking for every mistake based on IMCI… Afterward, advised us on how to do better" *(IDI 7B*, *PHC facility manager*).

Nurses from the district used the IMCI guidelines as a benchmark to assess the consultations; however, the facility and ward managers did not have a standard for monitoring SAM diagnosis.

#### Limited real-time consensus

The dietician-doctor communication on SAM diagnosis at hospitals was provided in writing, limiting engagement between these professionals to reach a consensus.

***Observations*:** doctors and dieticians had separate ward rounds. They made notes with different views on the same paper for four files (Files 1A, 3A, 1B, 3B), and consecutively for three days without consolidating in a Non-NHI file (File 1B).

The absence of real-time collaboration in SAM diagnosis between doctors and dieticians also led to doctors treating children for SAM while dieticians were recommending treatment for moderate acute malnutrition (MAM) or co-morbidities only.

### Limited adherence to hypoglycaemia prevention guidelines

Limitations in preventing hypoglycaemia and related factors are portrayed in Table 6.

**Table 6:** Limited adherence to feeding guidelines.

Broad Themes	Practices Sub-Themes	Factors Sub-Themes
Limited adherence to feeding guidelines to prevent hypoglycaemia	◾ Not feeding RUTF at PHC and EMS levels ◾ Facility-made RUTF without mineral mix ingredient	◾ Financial constraints ◾ Non-inclusion of RUTF in the national Essential Medicine List ◾ Clinical scope of practice limits for EMS personnel

Feeding to prevent hypoglycaemia was only administered for 1/19 children served at PHC facilities, not reported during transit, and involved feeding F75 or formula for 17/21 children at the hospital level. Lack of feeding at PHC facilities delayed care:

"Severely malnourished children at the clinic should be fed starter formula immediately as per requirement…when they have taken nothing, the blood glucose goes down" *(IDI 2A*, *Doctor*).

Despite the IMCI recommendations to administer follow-up feeding while referring children, the ambulance personnel did not feed them.

“We don’t have to feed children; we just give oxygen and make sure the patient is transported to the hospital” *(IDI 4B*, *EMS personnel)*.

At the hospital level, feeding F75 and transitioning to F100 was practiced; however, the Non-NHI hospital used self-made formulas that lacked an essential ingredient–*mineral mi*x–while the NHI hospital only used self-made formulas during stock-outs.

"We prepare the F75 and F100 in our kitchen…I think children like it better than the already prepared one. All ingredients are available apart from the mineral mix, but the formula works" *(IDI 3B*, *Ward Manager)*.

The use of therapeutic food without mineral mix to prevent hypoglycaemia limited the quality of the formula feed.

### Factors influencing unavailability and quality of therapeutic food

#### Financial constraints

Financial constraints partly contributed to the unavailability of F75 at the PHC level and the preparation of self-made therapeutic food without mineral mix and stock-outs at NHI hospitals.

“There is no F75 at clinics because of budgetary issues; we do not have enough to keep things at that level” *(IDI 40B*, *Dietician)*.

“The mineral mix is expensive, so in times of scarcity, we prepare feed without it….the scarcity is mainly due to budgetary constraints” *(IDI 3A*, *Dietician)*.

#### Non-inclusion of RUTF in the national drug list

The lack of a national mandate to make RUTF and mineral mix available also influenced whether they were readily available.

"The essential drug list for South Africa does not include mineral mix and ready-to-use therapeutic food, that is why they are not readily available" *(National Child Health Committee Feedback Session-Dietician)*.

The drugs that are included in the drug list are locally available, and at reasonable prices, thus, the non-inclusion of RUTF and the mineral mix was possibly due to their high price.

#### Scope of emergency services practice

The lack of feeding F75 during referral was due to the unavailability of an advanced cadre of emergency services personnel in the study subdistricts:

“We don’t have advanced life support emergency personnel which is the highest qualification… that’s a huge burden on the health system, especially on the emergency because they are allowed to give advanced care …" *(IDI 9A*, *EMS Manager)*.

The scope of practice of the EMS personnel at study subdistricts did not include feeding.

### Inconsistent adherence to SAM management steps

Table 7 portrays inconsistent adherence to specific SAM management steps and related factors.

**Table 7:** Inconsist ent adherence with SAM management steps.

Broad Themes	Practices Sub-Themes	Factors Sub-Themes
Inconsistent adherence to SAM management steps	◾ Inconsistent antibiotic, rehydration and vitamin A prescription	◾ Lack of skills ◾ Unavailability of summarised guidelines ◾ Irregular and poor monitoring and supervision

Despite being required for all children before referral, 6/19 were given antibiotics and 7/19 received vitamin A. Signs of dehydration led to rehydration of 3/19 children before the referral process.

"Most of the kids have diarrhoea, so before I refer, I give ORS… If it is not possible, I have to insert an IV line" *(IDI 19B*, *PHC Nurse)*.

"Before a referral, I give the child multivitamins and Philani [fortified porridge to be prepared at home]…other treatments are for the hospital” *(IDI 9B*, *Mobile Nurse)*.

EMS personnel mainly ensured circulation through rehydration during referrals:

"…as an ECT [emergency care technician], I know that I have to ensure that I put a drip on that child [with malnutrition] to give him the electrolytes… if the poor child has diarrhoea, he or she is losing the fluids" (IDI 4, EMS Supervisor).

Despite the IMCI recommending follow-up antibiotics during referrals, they were not administered.

At the hospital level, the following WHO steps for managing SAM were *inconsistently* implemented:

✓ The prevention of dehydration was intravenous for 13/21 children, while the recommended oral rehydration solution (ORS) was only prescribed to 2/21 children.
✓ Antibiotics were used to treat infections for 16/21 children. Antibiotics prescriptions varied per subdistrict; the NHI hospital only prescribed amoxicillin while the Non-NHI hospital prescribed a variety of antibiotics (including Ampicillin, Bactrim, and Gentamicin).
✓ The correction of micronutrients varied per child as zinc was prescribed for 13/21, folic acid for 3/21 and multivitamins for 9/21 children.
✓ The management steps that were least complied with by health workers/professionals were balancing electrolytes using potassium (1/21children), recording stimulation (1/21children children) and planning follow-up (3/21children). The recorded follow-ups were from the NHI hospital.

However, these hospital findings need to be viewed with caution due to missing or unrecorded rehydration measures for 2/21 children, infection treatment for 5 /21 children and micronutrient correction for 1/21 child.

At NHI hospital doctors skipped some steps; for instance, infections were treated for 4/7 and micronutrients were corrected for 3/7. Contrarily, Non-NHI hospital doctors did better. For instance, infections were treated for 12/14 children and micronutrients were corrected for 10/14 children.

"First, if the child with malnutrition comes, we follow those steps [participant points at a chart]…that’s what we call the ten steps of the management of acute malnutrition" *(IDI 2B*, *Doctor)*.

In implementing the following-up step, the Non-NHI hospital was inconsistent while the NHI hospital consistently utilised a well-structured system:

“The previous senior dietician started a follow-up system involving booking patients on a certain day… then you SMS (text message) a reminder…if they don’t come, I call, if I fail, I send a CHW through their leader. I release cases whenever I feel malnutrition is resolved" *(IDI 3A*, *Dietician)*.

### Factors influencing inconsistent implementation

#### Lack of skills

The inconsistent compliance with SAM management guidelines was partly due to the general doctors’ and nurses’ limited skills compounded by continuous rotations from paediatric consultation rooms and wards alongside the absence of skill development programs. At 5/9 facilities [3 NHI and 2 Non-NHI], general doctors and nurses were rotated to various departments within the facility. The long-serving nurses with uninterrupted service in SAM departments had the opportunity to gain expertise in SAM management.

“The nursing staff in paeds [paediatric ward]… those that have been here for a long time they are very experienced, they assist us [doctors] in planning SAM management” *(IDI 2B*, *Doctor)*.

To address skill deficiency, the Non-NHI hospital conducted ongoing skill development programs including refresher training for older staff and orientation for new staff. Additionally, the Non-NHI paediatric ward doctor was undergoing a mentoring program with senior paediatricians who kept him updated on current SAM management requirements.

#### Summarised guidelines

Non-NHI hospital’s better implementation of SAM guidelines was partly because of using a point format summarised WHO chart with ten steps of managing SAM which were strategically pasted on facility walls.

"…doctors on call from other departments are likely to jump other steps; therefore, this summary puts them in line…When I notice problems, I refer them back to them [summary of 10 steps]" *(IDI 3B*, *Ward Manager)*.

The summarised guidelines served as mandatory regulation for doctors serving children and an implementation monitoring tool.

#### Irregular and inadequate monitoring

The non-compliance to guidelines was also partly due to lack of accountability to supervisors compounded by the absence, irregular or poor supervision structures:

"The last time I was supervised was in 2008; now they [subdistrict office] are short of staff to come to supervise" *(IDI 9B*, *Mobile Nurse)*.

"I don’t think we are adequately supervised …we used to have a clinical manager, but now we [doctors] appoint [a clinical manager] within ourselves; since we are at the same level, there is no fear of something happening if we don’t do well" *(IDI 7A*, *Doctor)*.

In six sites (NHI hospital, NHI Clinic1, NHI Clinic2, Non-NHI Clinic2, and EMS stations), supervisors perceived themselves as efficient, yet their capacity, qualifications and experience can be or were scrutinised:

"I’ve never received any formal management training… I’m just a professional nurse with general training, but I manage my work… after the doctors have done their doctors’ rounds, I check whether everything that was supposed to be done was done" *(IDI 3A*, *Ward Manager)*.

"I don’t know how the manager was appointed, but I am more qualified and experienced than him, I am not sure how he can supervise me" *(IDI 18A*, *PHC nurse)*.

"The thing is you are qualified as a level six emergency care technician, and then your manager is on an intermediate level, so he can’t supervise you" *(IDI 9A*, *EMS personnel)*.

The supervisors were either junior to their supervisees or in acting positions to effectively oversee others.

### Unavailable and inconsistent documentation

PHC facilities lacked or inconsistently documented SAM management. All NHI facilities had under-five children files, while in the Non-NHI subdistrict, only the hospital had under-five children files. NHI subdistrict PHC facilities referred children with a RHB, referral letter and an IMCI form, while the Non-NHI subdistrict PHC facilities only used RHBs:

"Referral letters are for adults; we have a RHB for children…usually, we do not keep a copy; the caregiver keeps the book" *(IDI 7B*, *PHC Manager)*.

Using RHB only influenced the recording of less information while the use of the IMCI form and referral letter enhanced the recording of more details including diagnosis and detailed account of pre-referral. However, some IMCI forms were not completed, thus lacking some information. In addition to written communication, three PHC facilities made pre-referral calls:

"If a child’s condition deteriorates after managing according to the IMCI, …I call the doctor who can say *‘give him this and that while you are still waiting for the ambulance’…*" *(IDI 18B*, *PHC Facility Manager)*.

The use of phone communication enhanced collaborative decisions on pre-referral care and whether to refer.

### Factors influencing documentation problems

Unavailability of subdistrict-specific referral policy, structure and meetings partially influenced documentation problems. Non-NHI subdistrict referral documentation inefficiency was partly because of reliance on the provincial referral policy which was general. Contrarily, the NHI subdistrict used a subdistrict-specific referral policy which was kept at each facility and also conducted referral meetings.

"We do have referral meetings with them [hospital level] to say, *‘guys*, *please let’s come up with a strategy to resolve the issue of miscommunication during the referral of our client’* "*(IDI 10A*, *PHC Manager*).

To achieve an NHI call to strengthen the subdistrict referral system, the specific referral policy and subdistrict-wide meetings enabled dialogue and the likely development of strategies to strengthen referral processes in the NHI subdistrict.

## Discussion

This study has found discrepancies in SAM diagnosis, non-compliance to some SAM management guidelines, variations of referral mechanisms across subdistricts, contradictory documentation within records, and restricted continuation of care. The factors influencing these practices include inadequate clinical skills; inconsistent supervision and monitoring; unavailability of subdistrict-specific referral policies and operational structures; and suboptimal national policies on therapeutic food. I acknowledge that this study was conducted seven years ago and some aspects may have changed. However, the findings are still useful and can not only inform new research but also policies related to managing children with SAM across all levels of the South African sub-district referral system.

This study found that anthropometric assessments were incomplete in study subdistrict, some health workers often neglected the assessment of weight for height, MUAC and oedema. This is consistent with other studies that report the missing or exclusion of children with SAM due to wrongly executing or skipping SAM diagnosis indicators [11,17,20]. Considering only 40% of cases are diagnosed as SAM by MUAC or WZH alone, it is advisable to always use both [16]. In addition, checking oedema is also critical. The faulty diagnosis was mostly from actor-related problems including a lack of skills by general nurses or doctors and a lack of real-time consensus with other cadres of health workers to confirm SAM diagnosis. The lack of diagnostic skills to detect SAM with complications by general doctors and nurses is supported by studies in SA and Gambia, which further recommend training and supportive supervision [17,20]. At the hospital level where dieticians also diagnosed SAM, general doctors’ consolidation with dieticians’ diagnosis could have resolved discrepancies. Doctors are often regarded as having more power in making clinical decisions; however, it should be acknowledged that dieticians are also a reliable authority in SAM diagnosis and management [19].

One of the key SAM management omissions by health workers in this study was the failure to administer therapeutic food to prevent hypoglycaemia at PHC facilities or administering therapeutic food without the mineral-mix ingredient. The unavailability of therapeutic food or ingredients was due to budgetary constraints. Previous research has acknowledged that not all ingredients for therapeutic food are locally available [40]. Findings from this study are consistent with other studies conducted in SA, which reported that self-made therapeutic food lacked mineral mix [20,41]. During the SA piloting of the WHO 10 steps in 1998, manufacturing the mineral mix was commissioned to a particular pharmacy [28]. The unavailability of cheap therapeutic food and its ingredients in SA is partly due to it not being included in the SA National Essential Medicine List, which enables easy and affordable access to medicine and related supplies. In this case, subdistricts are affected by the absence of structures to enable easy administration of therapeutic food within national health department. On the contrary, a study conducted in India reported that the Ministry of Health ensured that ingredients like vitamins and mineral premix were available and locally produced. It also commissioned small manufacturing units to produce therapeutic food in settings with a high SAM prevalence [42]. At the global level, UNICEF, as a major supplier of RUTF, tracks the supply chain by focusing on supply and demand; local supply chains are urged to stock, borrow from other facilities or use alternative strategies [43]. If countries with high SAM incidences are not accessing RUTF from UNICEF, governments are obligated to strengthen supply chains or create an enabling environment for supporting alternative options like local production.

While unstructured documentation systems hindered the continuity of SAM care in the study setting, parts of Ghana, Brazil, and Saudi Arabia have experimented and adopted the use of electronic information systems, which have enhanced care collaboration across health workers and facilities [44–46]. Whether electronic or paper-based, information systems encourage knowledge sharing and resolving medical challenges because they contain patient history, diagnosis, and care administered for health workers initiating and receiving referrals [47,48]. Operationally, referral policies should portray pre-referral arrangements (calls, documentation and care), instructions on transit care, instructions for referral facilities to either investigate, treat and refer back patients for rehabilitation [48]. Revising referral system policies to address SAM in the study setting will require locally revising pre-referral arrangements, instruction on care requirements for emergency services and rehabilitation approaches, which can be adapted from the NHI subdistrict follow-up structure. Since 2008, the SA health department disseminated a reviewed national healthcare policy in 2020; the policy is meant to ensure timely referral and access to comprehensive healthcare services with the aim of maintaining the continuum of care [49].

Findings from this study link poor SAM management to health workers’ limited skills due to general training, frequent rotations and lack of on-job training, as similarly found in studies conducted in Ghana and Ethiopia [25,50]. The specialist knowledge of SAM services has been attributed to nutrition courses in college, length of service, and ongoing post-college refresher courses [25,51]. Through this study, we recommend continuous mentorship of general doctors at district hospitals by paediatricians at the tertiary hospital because the strategy was perceived effective in improving clinical skills in SAM management at NHI hospital. In addition to continuous training, mentorship and restricting rotations, a study on IMCI recognised the need to fill a gap in defining paediatric staffing needs [18]. For instance, the need to strengthen the skills of general doctors and nurses in the SA context is partly due to a decrease in specialists and an increase of generalist doctors (and nurses) in the SA public sector [52]. A gap in the specialist training model has also been noted; there is a lack of community paediatric skills due to university courses that are not aligned with SA’s community needs [52]. Therefore, a proposal to train a sub-speciality in Community Paediatrics and Child Health to curb skill shortages in rural SA was made to the government in 2012 [52]. The multi-level approach involving universities and the government has the potential to address skills inadequacy [19].

Another key factor that influenced discrepancies in diagnosis and non-compliance to management guidelines was the implementation of pre-defined or facility-developed support aids/tools which empowered implementers through internalising the implementation process [19]. This study highlights the importance of the summarised WHO 10 steps for inpatient treatment of severely malnourished children as both an essential implementation support and monitoring tool. Similar supplements for SAM diagnosis have the potential to enhance adherence. However, the IMCI form meant to supplement diagnosis is considered time-consuming by health workers [18]. Therefore, guidelines’ supplements should abide by the eighth commandment for effective clinical decision support, which asserts that attaining clinicians’ enthusiasm to use guidelines is achievable upon substantially condensing and simplifying their guidelines to fit a single screen (or page) to serve as job aids [53]. The presence of job aids has the potential to enhance clinical governance. Clinical governance, in this case, involves a mechanism to ensure that high clinical standards are maintained, and the quality of services continuously improved [54].

The managers’ limited competency in supervision and lack of monitoring tools limited the implementation quality. These findings correspond with studies in 103 countries, which found that managers’ incompetence in managing and monitoring IMCI implementation was because they had similar training to implementing health workers and none on management of implementation practices [55,56]. Similarly, PHC facility managers in Kenya also attributed their limited managerial skills to being appointed without training and learning on the job [57]. The manager’s role is meant to concentrate on effective oversight, regulations, accountability and attention to systems designs [58]. Training managers on monitoring alone is incomplete if criteria for monitoring implementation do not accompany guidelines. The government of Nepal has successfully substituted two days of IMCI training with a managers’ training program to strengthen planning, supervision, and monitoring skills. However, equipping managers with real-time implementation monitoring criteria will enable the identification of aspects that work or do not work, and context-specific factors that enable or hinder implementation [59]. In the Eastern Cape Province of SA, combinations of efforts enable successful monitoring and evaluation. A nutrition team composed of a doctor, two senior nurses and academics monitor performance indicators and continually provide solutions for identified gaps [10,60]. While facilities in the study setting and Mpumalanga Province audit deaths, the academics monitor the implementation of guidelines every two months while matrons have implementation quality meetings weekly in the Eastern Cape Province [41].

### Strengths and limitations

Adopting a qualitative case study design that employed a maximum variation sampling enabled the comparison of two distinct subdistricts and their complexities as a whole [31]. However, being situated in two subdistricts of the North West Province limits the generalisability of findings to other locations. Nonetheless, the study can be replicated using a different population or conceptually replicated using different procedures on the same population [61].

The exploration of the implementation of SAM guidelines’ was limited due to difficulties in accessing patient files. Future studies should aim to source a representative sample of files to enable advanced analysis which may lead to the establishment of significant implementation gaps.

## Conclusion

To curb preventable SAM admissions and deaths, there is a need to address subdistrict referral systems’ variations, non-compliance to management guidelines, and discrepancies in SAM diagnosis. The importance of referral systems as a means of achieving the continuation of SAM care needs to be realised by locally developing or adapting SAM referral policies. The improvements should uphold essential steps to strengthen information systems. The discrepancies in SAM diagnosis and management can be prevented by equipping implementers with adequate skills, supervision by competent managers and availability of summarised job aids. Lastly, there is a need to advocate for national policy commitment to ensuring the availability of RUTF and mineral-mix at facilities.
